# Secondary cytoreduction surgery improves prognosis in platinum-sensitive recurrent ovarian cancer

**DOI:** 10.1186/1756-9966-32-61

**Published:** 2013-09-02

**Authors:** Xia Xu, Xiaoxiang Chen, Zhiqin Dai, Fei Deng, Junwei Qu, Jing Ni

**Affiliations:** 1 Department of Chemotherapy, Jiangsu Cancer Hospital, Nanjing, Jiangsu 210009, PR China; 2 Department of Gynecologic Oncology, Jiangsu Cancer Hospital, Nanjing, Jiangsu 210009, PR China; 3 State Key Laboratory of Bioelectronics, Southeast University, Nanjing 210096, PR China

**Keywords:** Epithelial ovarian cancer, CA-125, Clinical relapse, Cytoreductive surgery, Time to progression

## Abstract

**Background:**

There is no consensus regarding the secondary cytoreduction surgery (CRS) in recurrent ovarian cancer patients. The present study aims to determine the value of secondary CRS and the eligible subgroup for this procedure.

**Methods:**

96 platinum-sensitive recurrent ovarian cancer patients were recruited from Jiangsu Institute of Cancer Research between 1992 and 2011. Follow-up was conducted based on the surveillance protocol of MD Anderson Cancer Center. Cox proportional hazards model and log-rank test were used to assess the associations between the survival durations and covariates. Logistic regression analysis was used to explore optimal secondary CRS related factors.

**Results:**

Optimal secondary CRS was associated with time to progression (TTP) and overall survival (OS) in patients (p < 0.01 both). Optimal secondary CRS and asymptomatic recurrent were similarly associated with longer OS (median: 79.2 *vs.* 53.9 and 76.1 *vs.* 56.0 months with p = 0.02 and p = 0.04, respectively) and TTP (median: 13.9 *vs.* 10.5 and 19.3 *vs.* 9.0 months with p = 0.02 and p = 0.03, respectively) than counterparts. Optimal initial CRS (p = 0.01), asymptomatic recurrent (p = 0.02) and longer progression-free survival duration (p = 0.02) were the independent indicators of optimal secondary CRS.

**Conclusions:**

Optimal secondary CRS had survival benefit for platinum-sensitive epithelial ovarian cancer. Asymptomatic recurrent was one of the recruited factors for this procedure.

## Background

Epithelial ovarian cancer (EOC) is the fifth most common cause of cancer mortality in United States and Chinese women [1,2]. The standard primary treatment paradigm of EOC includes optimal primary cytoreductive surgery (CRS) followed by platinum/paclitaxel based chemotherapy. Although more than half of EOC patients results in a complete clinical response (CCR) through initial therapy, achieving complete cure is infrequent. In fact, about 75% EOC patients develop recurrent disease within 2 years and the mean 5-year survival rate following the radiological defined recurrence is less than 10% [3]. The management of recurrent diseases is one of the key topics and is less clear than that of primary EOC. Salvage chemotherapy and secondary CRS were the two major therapeutic choices for the recurrent ovarian cancer. Despite the significant progress in chemotherapy and biological agents, surgery is still the cornerstone of recurrent patients’ management. Secondary CRS may be possible to improve the chance of objective response and/or a longer interval of second remission. Exploring the potential beneficial subpopulation and selection criteria of these two treatments is indispensable.

Observational studies have explored that secondary CRS may improve the survival duration of recurrent EOC patients. At least in platinum-sensitive recurrent EOC, the optimal secondary CRS shows a certain positive significance [4-9]. In addition to the potential benefit of secondary CRS, defining the specific population that might best benefit from this surgery is equaled important. Secondary CRS should be benefit to carefully selected patients who meet certain criteria amenable to complete gross resection was general accepted. Presently, identifying the eligible subgroup for the potentially morbidity-inducing procedure remains a clinical challenge and in practice, gynecologic oncologists use their own qualifying criteria will vary from one to others. The series trials of DESKTOP identified an independently predictive score for complete resection comprehensive of good performance status, complete resection at primary surgery, and the absence of ascites [10,11]. Zang et, al. found a patients’ selected model for optimal secondary CRS in recurrent ovarian cancer includes FIGO stage, residual disease after primary surgery, progression-free interval, ECOG performance status, CA125 at recurrence, ascites at recurrence. Our previous study revealed that rising CA-125 levels optimized the secondary CRS in asymptomatic recurrent EOC [12]. Other factors predict surgery outcome of secondary CRS includes progression-free survival (PFS) from primary treatment to recurrence, and number of recurrent tumors [13].

In the present study, we retrospectively evaluated platinum-sensitive recurrent ovarian cancer patients who underwent secondary CRS. Factors affecting the outcome of secondary CRS were analyzed to reveal those who potential benefit with the opportunity for this procedure.

## Methods

### Study population

Present research was approved by Jiangsu Institute of Cancer Research (JICR). We identified 96 platinum-sensitive recurrent EOC patients at JICR from clinical stations between January 1, 1992 and January 1, 2011. Among them, 43 cases underwent secondary CRS. Those who did not undergo the standard first line treatment and achieved CCR or platinum resistance recurrent were excluded. Secondary CRS as a selective procedure was performed in patients with good performance status and intended purpose of tumor reduction. After primary therapy, the routine follow-up protocol was conducted as described previously. The Response Evaluation Criteria in Solid Tumors (RECIST) criterion was used to assess treatment response and tumor progression [14-16]. The clinicopathological data including the histological type and grade of the tumor [17,18], stage of the disease [19], volume of ascites, time to progression, management of primary and recurrent disease, and time of death or last follow-up. Pathological diagnoses of recruited cases were reviewed by two JICR pathologists, namely, X. Xu and L. Hou.

### Definition of clinical response and surveillance

The definition of CCR includes the absence of tumor-associated clinical symptoms and residual tumor on the physical examination, EOC-negative imaging study results and a serum CA-125 concentration below the upper limit of the normal range (ULN = 35U/mL) in the current study. Clinical recurrent was identified as the occurrence of any new measurable lesion through imaging studies or clinical examination [15]. Patients underwent neoadjuvant chemotherapy followed by interval CRS. Platinum-sensitive recurrent was generally referring to the progression of the free interval at least 6 months from the completion of primary therapy. According to most of the gynecologists, secondary CRS is defined as an debulking procedure performed at some time remote (generally disease free interval of more than 6 months) from the completion of primary treatment with the intended purpose of tumor reduction. The criterion of optimal CRS was the threshold of residual tumor ≤ 1 cm or macroscopic free and suboptimal debulking was defined as more than 1 cm of nodules left. The overall survival (OS) duration was defined as the time from the disease diagnosis to death or last follow-up. PFS was the length of time during and after initial therapy wherein the patient’s condition does not worsen. Time to progression (TTP) was a measure of time from radiological defined relapse to the disease starts to get worse in present study.

### Statistical analysis

Cox proportional hazards model was used to assess the relationship between the clinical characteristics and the OS and TTP. Step-wise regression was conducted to build the multivariate models. The log-rank test was used to assess this relationship. Logistic regression analysis was used to explore optimal secondary CRS related factors. The p values < 0.05 was considered statistically significant. All analyses were conducted using the SPSS statistical software program (version 18.0; SSPS Inc, Chicago, IL).

## Results

### Patient characteristics

The clinicopathological characteristics of all patients included in the present study were given in Table 1. High-grade and low-grade primary EOC were 83 (86.5%) and 13 (13.5%), respectively, and serous carcinoma cases was 67 (69.8%). Median follow-up time was 37.6 months (interquartile range, 20.2 months to 69.0 months) in the living patients at the beginning of our analysis. The recurrent patients underwent secondary CRS were reported experiencing pain (2 patients), gastrointestinal dysfunction (8 cases), and/or mass effect (7 cases) and others (7 cases). Nineteen cases were asymptomatic biochemistry recurrence. In all 96 patients who underwent platinum-sensitive clinical recurrent, 48 (50.0%) patients were CA-125 indicated asymptomatic relapse.

**Table 1:** Patient characteristics of the study population

**Characteristic**	**Percentage (%)/Median (range)**	
**Age (years)**	61.6	(26–82)
**Baseline CA-125 level (U/mL)**	582	(5–24260)
**Nadir CA-125 level (U/mL)**	10	(3–35)
**Histology**		
Serous	67	(69.8)
Endometrioid	10	(10.4)
Clear cell	8	(8.3)
Mucinous	4	(4.2)
Transitional	3	(3.1)
Undifferentiated	3	(3.1)
Malignant mixed müllerian tumor	1	(1.0)
**Grade**		
Low	13	(13.5)
High	83	(86.5)
**Surgical residual**		
<1 cm	62	(64.6)
1–2 cm	3	(3.1)
>2 cm	17	(17.7)
Unknown	14	(14.6)
**FIGO stage**		
I	9	(9.4)
II	8	(8.3)
III	63	(65.6)
IV	14	(14.6)
Unknown	2	(2.1)
**Neo-adjuvant chemotherapy**	68	(70.6)
**Paclitaxel-based**	82	(85.4)

FIGO the International Federation of Gynecology and Obstetrics.

### Survive related factors in platinum-sensitive recurrent ovarian cancer

Univariate Cox proportional hazards model revealed that FIGO stage, pathological grade, outcome of CRS, nadir CA-125 level, ascities and PFS were associate with OS and TTP in all patients (Table 2). Multivariate analysis revealed that grade, nadir CA-125 level, optimal secondary CRS, ascities and PFS were independent OS and TTP predictors in platinum-sensitive recurrent EOC (Table 3).

**Table 2:** Univariate analysis of survival-related characteristics in platinum-sensitive recurrent ovarian cancer

**Variable**	**TTP (OR 95% CI)**	**OS (OR 95% CI)**
**FIGO stage**		
I	1.00(reference)	1.00(reference)
II	1.25(0.57–4.31)	1.44(0.66–4.45)
III	3.09(1.53–8.36)	3.71(2.34–8.95)
IV	4.64(2.85–12.26)	4.96(2.51–11.14)
**Grade**		
Low	1.00(reference)	1.00(reference)
High	5.22(2.14–12.76)	4.02(1.95–10.33)
**Ascites**		
No	1.00(reference)	1.00(reference)
Yes	1.78(1.44–2.38)	1.94(1.48–2.27)
**Optimal initial CRS**		
Yes	1.00(reference)	1.00(reference)
No	6.07(2.50–15.91)	6.84(3.32–13.86)
**Optimal secondary CRS**		
Yes	1.00(reference)	1.00(reference)
No	5.28(1.86–16.93)	9.30(4.29–19.51)
**Neo-chemotherapy**		
Yes	1.00(reference)	1.00(reference)
No	1.19(1.04–1.57)	1.45(0.79–2.75)
**Paclitaxel-based chemotherapy**		
Yes	1.00(reference)	1.00(reference)
No	1.02(0.85–1.39)	1.35(0.83–2.01)
**PFS**	1.02(1.00–1.18)	1.13(1.07–1.30)
**Nadir CA-125**	1.02(1.00–1.03)	1.03(1.00–1.06)

*CRS* cytoreduction surgery; *OS* overall survival; *TTP* time to progression; *PFS* progression-free survival.

**Table 3:** Multivariate analysis of survival-related characteristics in platinum-sensitive recurrent ovarian cancer

**Variable**	**TTP (OR 95% CI)**	**OS (OR 95% CI)**
**Grade**		
Low	1.00(reference)	1.00(reference)
High	3.74(2.01–10.35)	3.83(1.69–9.47)
**Ascites**		
No	1.00(reference)	1.00(reference)
Yes	1.62(1.37–2.51)	1.76(1.43–2.36)
**Optimal secondary CRS**		
No	1.00(reference)	1.00(reference)
Yes	6.27(3.84–14.28)	8.21(2.37–28.60)
**PFS**	1.02(1.00–1.14)	1.10(1.04–1.36)
**Nadir CA-125**	1.02(1.00–1.02)	1.03(1.00–1.04)

**Figure 1:**
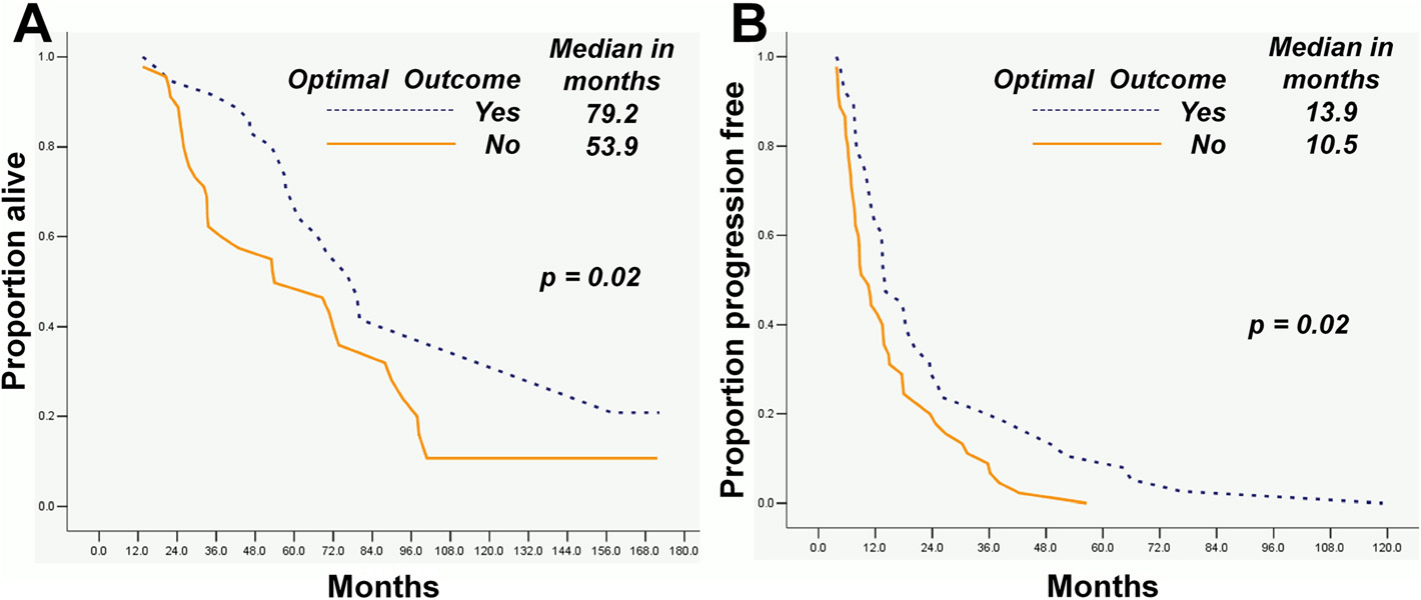
Patients who underwent optimal secondary CRS had longer OS and TTP durations than those who did not undergo (1A, 1B).

**Figure 2:**
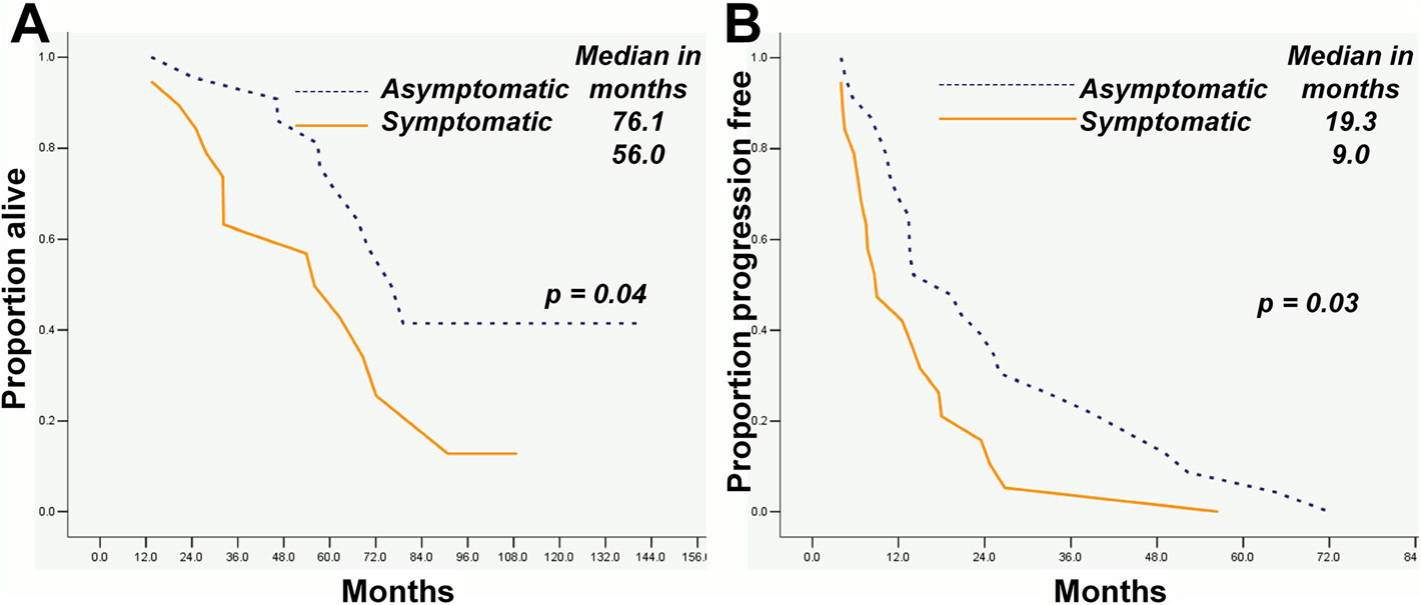
Symptomatic recurrent patients who underwent secondary CRS had shorter OS (A) and TTP (B) durations than asymptomatic ones (2A, 2B).

The OS and TTP durations of ovarian cancer patients who underwent optimal secondary were longer than those who did not undergo (p = 0.02 and p = 0.02 respectively; Figure 1A and B). In patients underwent secondary CRS, the OS and TTP durations of asymptomatic cases were longer than those of symptomatic ones (p = 0.04 and p = 0.03 respectively; Figure 2A and B).

### Optimal secondary CRS associated factors

To explore the potential factors related to optimal secondary CRS, we performed logistic regression analysis in platinum-sensitive recurrent ovarian cancer patients, we found that optimal initial CRS (p = 0.01), asymptomatic recurrent status (p = 0.02) and longer progression-free survival duration (p = 0.02) were the independent indicators for OS and TTP (as seen in Table 4).

**Table 4:** Logistic regression of optimal secondary CRS-associated factors in platinum-sensitive recurrent ovarian cancer

**Variable**	**Univariate**		**Multivariate**	
	**Exp(B)**	**Sig**	**Exp(B)**	**Sig**
Age	1.01	0.12	1.00	0.43
Ascites	1.40	0.02	1.33	0.15
Initial CRS	2.63	0.00	2.29	0.01
PFS	2.02	0.01	1.85	0.02
Recurrent status	1.96	0.00	1.52	0.02
Stage	1.25	0.00	1.20	0.19
CA-125 at recurrent	1.05	0.15	1.02	0.36

## Discussion

The high recurrence rate and the lack of effective treatments incurs therapeutic dilemma in the management of EOC. Presently, the standard care of recurrent EOC is salvage chemotherapy but not SCR for recurrence is considered to be incurable. The Secondary CRS is a treatment option for selected patients with recurrent EOC. Though being examined by several retrospective or nonrandomized prospective studies, the prognostic role and the utility criterion of secondary CRS still remain controversial [8,20-26]. One prospective study suggested that optimal secondary CRS was feasible for the most of patients with recurrent EOC and confers survival benefit while combined with salvage chemotherapy [26]. On the contrary, another study stated that secondary CRS does not improve PFS or OS in patients underwent initial optimal surgery [27]. Ongoing prospective multi-centers trials (DESKTOP III and Gynecologic Oncology Group Protocol 213) to probe the survival benefit of secondary CRS and second line chemotherapy in patients with recurrent EOC may help to settle disputes partly [28]. Other factors including performance status, preoperative and post-operative chemotherapy, histologic type, ascites, elevated CA 125 level and number of recurrent tumors at recurrence were reported to be prognostic factors [4,20,26,29]. In our series, tumor grade, ascites, nadir serum CA 125 level, optimal secondary CRS and progression-free interval were independent prognostic factors for TTP and OS.

It is generally believed that secondary CRS has a survival benefit in select platinum-sensitive patients with recurrent ovarian cancer. Who will benefit from or say will appropriate for secondary CRS is another important concern on this topic. Minimal residual tumor and longer progression-free interval were reported to indicate improving survival outcomes in most studies [5,8,30,31]. On the other hand, some studies found residual tumor and progression-free interval had no impact of on prognosis in recurrent EOC underwent secondary CRS [4,6,7,28,32]. Our previous study found that CA-125 indicated asymptomatic recurrent cases will benefit from optimal secondary CRS [12]. Zang et al. emphasized the number of recurrent tumors. They stated those patients with solitary lesions, no ascites at recurrence, achieved initial optimal surgical outcomes and survival benefit more easily for secondary CRS and further confirmed it in a large population more than one thousand cases [20,21,33]. Berek et al. reported that recurrent tumor size had an impact on survival while Park et al. denied the relationship between the size of the recurrent tumor and survival outcomes [5,29]. In our series, three major prognostic factors affected survival after secondary CRS: optimal resection after initial CRS, asymptomatic recurrent status and longer PFS duration after primary treatment.

Morbidity and mortality rates during perioperative period are also important issues when secondary CRS is considered in the management of recurrent ovarian cancer. Postoperative morbidity rates reported to be ranged from 5% to 35% in different trials [5,23,26,34]. In general, secondary CRS was considered to be a safe procedure in the management of recurrent EOC [5,35,36]. There was no operation related deaths in our series.

There are limitations to the present study. Firstly, unavoidable selection biases inherent to its retrospective design. CRS status, chemotherapy regimens and some additional salvage therapy may have reflected certain selected factors that may influence prognosis, though we eliminate the influence of consolidation or maintenance treatment by inclusion criteria. Secondly, given the long time follow up and the heterogeneity of therapy strategies used throughout the 23 years study period, including the emergence of new regimens such as paclitaxel based chemotherapy and targeted therapy and so on, it was impossible to unify the therapy strategy. Thirdly, the absence of unified recruited standard for secondary CRS and limited sample size were factors may also cause selection bias. Last but not nest, populations underwent secondary CRS was relatively young and healthy with a good performance status, and a high likelihood of endure postoperative chemotherapy. It cannot be translated to all recurrent EOCs until further studies with broader inclusion criteria are available. Evaluating patients from China with validation set from America may help to lessen this unfavorable effect.

In summary, in this study including patients from two centers with same recruited standard, we found that secondary CRS has survival benefit to selected patients. The recruited criterion included asymptomatic recurrent, optimal initial CRS and platinum recurrent with comparatively longer tumor free interval.

## Abbreviations

CCR: Complete clinical response; OS: Overall survival; PFS: Progression-free survival; TTP: Time to progression; OR: Odds ratio; CI: Confidence interval; EOC: Epithelial ovarian cancer; CRS: Cytoreductive surgery; ULN: Upper limit of normal; RECIST: Response evaluation criteria in solid tumors.

## Competing interests

The authors declare that they have no competing interests.

## Authors’ contributions

XX and XXC participated in drafting the full manuscript and writing of this manuscript. FD and JN partly participated in clinical study design, coordination and data analysis. ZQD, JN participated in collecting data, creating figures and tables. XX, FD and JN contributed by writing specific sections of this manuscript. CML and YBZ provided advice and participated in revising the manuscript. XXC participated in substantial contribution to conception and revising it critically for important intellectual content. All the authors in this manuscript have read and approved the final version.
